# Dendritic Cell Cancer Vaccines: From the Bench to the Bedside

**DOI:** 10.5041/RMMJ.10158

**Published:** 2014-10-29

**Authors:** Tamar Katz, Irit Avivi, Noam Benyamini, Jacalyn Rosenblatt, David Avigan

**Affiliations:** 1 Department of Hematology and Bone Marrow Transplantation, Rambam Health Care Campus, Haifa, Israel;; 2 Bruce Rappaport Faculty of Medicine, Technion, Israel Institute of Technology, Haifa, Israel; 3 Hematological Malignancies and Bone Marrow Transplantation, Beth Israel Deaconess Medical Center, Harvard Medical School, Boston, MA, USA

**Keywords:** Cancer vaccines, dendritic cells, hematological malignancies, immunotherapy

## Abstract

The recognition that the development of cancer is associated with acquired immunodeficiency, mostly against cancer cells themselves, and understanding pathways inducing this immunosuppression, has led to a tremendous development of new immunological approaches, both vaccines and drugs, which overcome this inhibition. Both “passive” (e.g. strategies relying on the administration of specific T cells) and “active” vaccines (e.g. peptide-directed or whole-cell vaccines) have become attractive immunological approaches, inducing cell death by targeting tumor-associated antigens. Whereas peptide-targeted vaccines are usually directed against a single antigen, whole-cell vaccines (e.g. dendritic cell vaccines) are aimed to induce robust responsiveness by targeting several tumor-related antigens simultaneously. The combination of vaccines with new immuno-stimulating agents which target “immunosuppressive checkpoints” (anti-CTLA-4, PD-1, etc.) is likely to improve and maintain immune response induced by vaccination.

## INTRODUCTION

In a large proportion of patients, hematological malignancies remain incurable with conventional chemotherapy. The most promising new cancer treatment approach is immunotherapy that harnesses the immune system to fight cancer by inducing or suppressing immune responses.

The earliest evidence for the role of the immune response against cancer cells was provided in the work by William Coley, who injected *Streptococcus pyogenes* to sarcoma patients in an effort to reproduce spontaneous remissions observed in cancer patients developing erysipelas. Ten percent of these incurable non-resectable patients responded.^1^ Similar promising results obtained in several other studies led to the use of bacilli Calmette–Guérin (BCG) for cancer immunotherapy, which continues to be employed to the present day as an effective therapy against superficial bladder cancer.^2^

The immune system has the ability to identify and eliminate tumor cells on the basis of tumor-specific antigens in the process of “immuno-editing” that includes three phases: elimination, equilibrium, and escape.^3^ At the elimination phase,^4^ tumor cells are efficiently detected and destroyed by the immune system. Tumor cells not completely eliminated by the immune system proceed to the equilibrium phase where the tumor persists but is not expanding. The escape phase begins when the balance between the immune response and the tumor moves towards tumor growth, which may be caused by immune exhaustion, inhibition, or occurrence of tumor cell variants that allow the tumor to evade the immune system.

The information regarding immune response to cancer remained controversial throughout the twentieth century; only with the development of mouse models has our understanding advanced significantly. For instance, a series of experiments demonstrated that mutant mice with severe combined immunodeficiency, lacking T and B lymphocytes, had a high tendency to develop lymphomas and carcinomas.^5^ Furthermore, other studies have shown that mice injected with irradiated tumor cells became protected against lethal viable cells of the same tumor.^6^

Accordingly, studies in humans reported a high incidence of lymphomas and other malignancies in immunocompromised states.^7^,^8^ Patients undergoing solid organ or bone marrow transplantation were found to be at an increased risk of developing post-transplant lymphoproliferative disorders (PTLD),^9^ and therefore discontinuation of immunosuppressive drugs potentially resulting in immunity reconstruction could contribute to successful management of these patients.

One of the earliest and most successful immuno-therapies developed for hematological malignancies was hematopoietic stem cell transplantation (HSCT). While this procedure is associated with significant morbidity and mortality, it has been shown to prolong long-term disease-free survival as well as overall survival and can be curative in a subset of patients. This efficacy of HSCT is attributed to the graft-versus-disease effect mediated by allogeneic donor T cells.^10^ Another demonstration of the impressive immune reaction in the HSCT setting is the donor lymphocyte infusion that can eradicate post-transplant disease relapse.^11^

## PRIMARY MECHANISMS OF IMMUNE ESCAPE

Although the immune system has a potential capability to recognize and attack cancer cells, tumor cells manage to escape immune recognition by employing different mechanisms which normally protect healthy tissues from autoimmune reactions. These mechanisms include inefficient processing and presentation of tumor antigens, up-regulation of negative co-stimulatory ligands which mediate T cell anergy,^12^ expansion of regulatory cells, and production of “immunosuppressive molecules,” such as transforming growth factor β (TGF-β),^13^ Fas ligand,^14^ and the immunosuppressive enzyme indolamine-2,3-dioxygenase (IDO).^15^ Tumor cells can also directly escape T cell recognition through down-regulating major histocompatibility complex (MHC) class I or disabling other components of antigen process.^16^

### Inefficient Processing and Presentation of Tumor Antigens

Recognition of tumor-specific antigens is mediated by selected MHC molecules. Tumor cells can directly escape T cell recognition through down-regulation of either MHC class I, or tumor antigen expression.

Moreover, cancer-induced defects in human leukocyte antigen (HLA) or in other antigen-processing molecules, like transporter associated with antigen processing (TAP), may also contribute to this immune escape. Additionally, cancer cells often induce secretion of “immunosuppressive” factors which interfere with dendritic cell maturation and function, leading to an inefficient T cell activation against tumor cells.

### Inhibitory T Cell Pathways

The T cell receptor co-stimulatory pathways are important immune checkpoints involved in maintaining homeostasis of the immune system by regulating T cell activation.

One of the significant immune checkpoint receptors is cytotoxic T-lymphocyte associated antigen 4 (CTLA-4) expressed on T cells which down-regulates T cell activation aiming to limit damage to self-tissues.^17^ The expression of CTLA-4 on the T cell surface reduces the activation of T cells by competing with the T cell co-stimulatory receptor CD28 in binding to identical ligands CD80 and CD86 and delivering inhibitory signals to the T cell.^18^ Upon binding CTLA-4 arrests T cell activation by down-regulation of CD4+ T helper cells and increase in immunosuppressive activity of T regulatory cells.

Tumor cells can exploit this pathway by expressing ligands for the CTLA-4 receptor on their surface. Blockade of CTLA-4 with specific monoclonal antibodies can shift the immune system balance toward T-cell activation, leading to tumor rejection. Both retrospective and prospective phase II and III studies have recently demonstrated a significant antitumor activity of the anti-CTLA-4 blocking antibody ipilimumab in advanced melanoma.^19^ The response rate in patients with advanced-stage disease who failed previous therapies and had no other potential therapeutic opportunities approached 40% and was translated into an improved survival.^20^,^21^ Interestingly, response was accompanied by increase in lymphocyte count and a decrease in regulatory T cells (Tregs).^21^

Another key checkpoint pathway mediating tumor-induced immune suppression is the programmed death-1 (PD-1); PD-1 is a cell surface inhibitory receptor expressed on T cells, B cells, monocytes, and natural killer T cells, following activation.^22^ It has two ligands: PD-L1 (B7-H1) and PD-L2 (B7-DC), both expressed on antigen-presenting cells (APCs).^23^ The ligand PD-L1, thought to be the main mediator of PD-1-dependent immuno-suppression, is also expressed on some non-hematopoietic cells. The interaction of PD-1 with its ligand inhibits T-cell receptor signaling, down-regulates the expression of some antiapoptotic molecules, and influences the cell cycle.^24^ The PD-1 pathway is an important regulator of induction and maintenance of peripheral tolerance involved in preventing tissue damage in chronic inflammation.^25^

The PD-1 pathway may have a key role in the interaction of tumor cells with the host immune response, and tumor cell PD-L1 expression may serve as a mechanism of adaptive immune resistance.

The ligand PD-L1 has been reported to be expressed on many different tumor cells.^24^ High PDL1 expression, at least in the solid tumor scenario, appears to correlate with increased tumor aggressiveness and high risk of death.^26^,^27^

Tumor-infiltrating lymphocytes (TILs) from patients with cancer, typically expressing PD-1, are characterized with an impaired antitumor functionality.^28^

Blockade of either PD-1 or its ligands with a specific monoclonal antibody enhances T-cell effector function, including cytolytic activity against tumor cells. Immune effects of the blockade have been shown in a variety of preclinical^29^ and clinical studies in both solid tumors (e.g. melanoma, lung cancer, etc.)^30^ and hematological malignancies.^31^ A recent study, investigating the safety and efficacy of anti-PD-1 antibody (Ab) in conjunction with rituximab in patients with relapsed follicular lymphoma, reported an encouraging safety profile and anti-lymphoma activity, with an overall response rate of 66%, including 52% complete remissions.^31^

### Regulatory Immune Cells

The tumor microenvironment is controlled by Tregs and other cell populations like myeloid-derived suppressor cells (MDSC) that create an immunosuppressive microenvironment and suppress antitumor effector T cells. Classic regulatory T cells are thymus-derived CD4^+^CD25^+^FOXP3^+^ T cells, which are responsible for inducing and maintaining peripheral tolerance through suppressing immune responses.^32^ Tregs also play a major role in tumor surveillance, suppressing an antitumor response both in tumor bed and systemically. Tregs are recruited to tumor sites, where they suppress anti-tumor cytotoxic responses. As most tumor antigens are self-antigens, Treg-mediated suppression has been proposed as a potential mechanism explaining the failure of antitumor immunity. Indeed, an increased number of Tregs in peripheral blood and tumor bed, often reported in both solid and hematooncological cancers, is associated with a worse prognosis.^33^–^35^

Depletion of Tregs or inhibition of their suppressive activity can enhance tumor immunity. This may be achieved using monoclonal antibodies (mAb) specific for cell surface molecules (CD25, Toll-like receptor, CTLA-4, GITR, OX40, and folate receptor 4) that are predominantly expressed by Tregs or specifically able to modulate Treg function.^33^ For instance, removal of Tregs by anti-CD25 mAb or toxin-conjugated anti-IL-2 (denileukin difitox) facilitates the activation and expansion of effector T cells that inhibit tumor growth in rodents. Since CD25 expression is also induced in activated effector T cells and IL-2 is required for the expansion of CD8^+^ T cells, treatment with anti-CD25 mAb or denileukin difitox may concurrently dampen effector T-cell responses.^36^

Preclinical data suggest Treg depletion to promote tumor regression.^37^–^39^ Development of new strategies aiming to attenuate selectively Tregs’ immunosuppressive effect in the tumor micro-environment is needed.

As blockade of CTLA-4 is known to abrogate the suppressive activity of Tregs and improves tumor immunity, the combination of anti-CTLA-4 mAb and anti-GITR mAb elicits a more potent antitumor response causing rejection of advanced stage tumors than does either mAb alone.

Another suppressive subset of cells is the heterogeneous population of MDSC that are expanded in cancer and have the capacity to suppress the immune response; MDSC are generated in the bone marrow in response to cancer-derived factors and are recruited to the tumor site by CCL2, CXCL12, and CXCL5.^40^ The MDSCs suppress the activation of T effector and natural killer cells and induce expansion of Tregs.

## VACCINE THERAPY FOR MYELOMA: REVERSING TUMOR-MEDIATED IMMUNE SUPPRESSION

The development of multiple myeloma is associated with progressive immune dysregulation that promotes tumor growth and resistance.^41^,^42^ The immunologic milieu is characterized by the diminished activity of antigen-presenting cells and loss of effector cell function including deficiencies in T and natural killer (NK) cell function. Myeloma cells present antigens in the absence of co-stimulation and inflammatory signals, resulting in the inactivation of potentially reactive T cell populations. As such, antigens that are aberrantly expressed by the myeloma clone are unrecognized.^43^,^44^ Investigators have sought out strategies to reverse tumor-mediated immune suppression such that malignant cells are designated as foreign by immune-based mechanisms and eliminated. One such approach is the use of tumor vaccines to present tumor antigens effectively in the context of immune-activating signals. A primary strategy is through the use of potent antigen-presenting cells known as dendritic cells (DCs) that constitutively express co-stimulatory molecules and inflammatory cytokines necessary for the primary activation of immunity.

Dendritic cells represent a diverse network of antigen-presenting cells that play a prominent role in mediating immune responsiveness.^45^,^46^ Circulating DC populations have been identified as myeloid and plasmacytoid in origin, characterized by the expression of CD11c and CD123, respectively. Myeloid DCs exhibit functional deficiencies in patients with myeloma that may impact their ability to elicit immunologic responses.^43^ Plasmacytoid DCs have been identified as stromal elements in myeloma that help to mediate tolerance. In contrast, *ex vivo*-generated DCs from patients with myeloma exhibit a functionally active phenotype characterized by expression of co-stimulatory molecules and stimulatory cytokines and may serve as a vehicle for tumor vaccines.^47^,^48^ Strategies to load tumor antigens include pulsing with peptides, proteins, or lysates, electroporation with tumor onto DCs including the use of whole-tumor cell or antigen-specific RNA, tumor-derived apoptotic bodies, transduction with viral vectors expressing tumor antigens, and whole-cell fusion between DCs and myeloma cells.^49^–^55^

## SINGLE ANTIGEN APPROACHES

Myeloma-associated antigens have been identified that serve as potential targets for cellular immunotherapy. The idiotype protein represents a myeloma-specific antigen represented by the unique immunoglobulin gene arrangement of the malignant plasma cell.^56^–^61^ Idiotype-based vaccines potentially induce a highly selective immune response but are potentially limited by uncertain immunogenicity and the challenge of isolating the unique M protein of each patient. In previously reported studies, vaccination with the idiotype protein in conjunction with GM-CSF or IL-12 was associated with antigen-specific T cell responses that correlated with improved outcomes.^62^ Responses have also been observed following vaccination with DCs pulsed with the idiotype protein and exposed to CD40L to induce maturation.^63^,^64^

Several shared antigens have been identified in myeloma cells that are uniquely or aberrantly expressed and serve as potential targets for immunotherapy. These include MUC1, WT1, PRAME, CYP1B1, and HSP96.^65^–^69^ A peptide-based vaccine for WT1 administered with immune adjuvant has been shown to elicit immunologic response in patients with hematological malignancies and a decrease in measures of disease.^70^ In a more recent study, WT1-specific T cells were detected in patients who had undergone allogeneic transplantation and correlated with durable remission. The cancer testis antigen, NY-ESO, demonstrates increased expression by plasma cells in the setting of advanced disease, creating an appealing target for immune-based therapy.^71^ Repetitive stimulation with DCs pulsed with an NY-ESO-derived peptide elicits a strong cytotoxic T lymphocyte (CTL) response *in vitro*, demonstrating an activated phenotype capable of lysing primary myeloma cells. Several other peptides which are highly expressed on myeloma cells and are important in the pathogenesis of the disease have been identified as potential immunogenic targets. Heteroclitic peptides derived from XBP1 (X-box-binding protein 1), CD138 (syndecan-1), and CS1 were shown alone or in combination to induce the expansion of myeloma-specific T cells with the capacity to lyse tumor cells.^72^–^75^ A trial is currently underway in which patients with smoldering myeloma undergo vaccination with combined myeloma-associated peptides in the context of immune adjuvant.

## WHOLE-CELL APPROACHES

The use of whole-cell-derived antigens for vaccination may be advantageous by eliciting a broad polyclonal response that effectively targets the heterogeneity of the myeloma cell population. In one example, DCs pulsed with myeloma cell lysates induce myeloma-associated immunity, although the clinical efficacy was uncertain.^47^ Other strategies that have been pursued include the use of whole-cell RNA, DNA, or apoptotic bodies for antigen loading onto DCs.^76^,^77^

We have developed a vaccine model in which patient-derived myeloma cells are fused with autologous DCs such that a broad array of myeloma antigens are effectively presented in the context of enhanced co-stimulation.^78^–^80^ In a murine adenocarcinoma model, vaccination with DC/tumor fusions protected animals from an otherwise lethal challenge of tumor cells. Most significantly, vaccination was able to eradicate established disease in animals with advanced pulmonary metastases. Similarly, DC/multiple myeloma (MM) cell fusions were effective in a syngeneic murine myeloma model, and therapeutic efficacy was further enhanced by co-administration of IL-12. In preclinical human studies, fusion of DCs and MM cells elicited the expansion of activated T cells that potently lysed autologous myeloma cells *in vitro*. We demonstrated that cell fusion induces DC maturation as manifested by increased expression of co-stimulatory molecules and maturation markers.^81^ Vaccine efficacy was further enhanced by exposure to inflammatory signals such as Toll-like receptor agonists. Sequential stimulation of T cells with DC/tumor fusions and ligation of the T cell costimulatory complex with anti-CD3/CD28 results in the dramatic expansion of tumor-specific lymphocytes.^82^

A phase I clinical trial was completed in which successive cohorts of patients with advanced myeloma underwent vaccination with escalating doses of autologous DC/MM fusions.^83^ Patients had undergone a median of four prior treatment regimens. Dendritic cells were generated from adherent mono-nuclear cells cultured with GM-CSF and IL-4 and matured with TNFα. Autologous myeloma cell preparations were obtained from bone marrow aspirates of patients with at least 20% marrow involvement with tumor cells. Dendritic cells and myeloma cells underwent phenotypic characterization to identify markers unique to each population. Dendritic cells were subsequently fused with autologous myeloma cells by co-culture in the presence of polyethylene glycol. Fusion cells were quantified by determining the percentage of cells that coexpressed unique DC and myeloma antigens. Vaccine production was feasible with achievement of the planned dose escalation up to a dose of 4 × 10^6^ fusion cells. Patients underwent serial vaccination in conjunction with GM-CSF. Vaccine-associated toxicity consisted of transient grade 1–2 vaccine site reactions most commonly, while clinically significant autoimmunity was not observed. Biopsy of the vaccine bed demonstrated a dense infiltrate of CD8+ T cells consistent with T cell expansion occurring at the site of vaccination. Vaccination resulted in the expansion of myeloma-specific T cells in a majority of patients as manifested by the percent of CD4 and/or CD8 T cells expressing IFNγ following *ex vivo* exposure to autologous tumor lysate. Humoral responses directed against myeloma-associated targets were documented by SEREX analysis. Of note, 66% of patients demonstrated a period of disease stability ranging from several months to greater than 2 years after vaccination.

## VACCINATION IN CONJUNCTION WITH AUTOLOGOUS TRANSPLANTATION

While vaccination induced anti-myeloma immunity in patients with advanced disease, we postulated that clinical efficacy was more likely in the setting of minimal disease in which tumor-mediated disruption of cellular immunity is less pronounced.

In animal models, the period of lymphopoietic reconstitution is associated with enhanced responsiveness to tumor vaccines due to the relative depletion of regulatory T cells and the increased presence of tumor reactive clones. Of note, vaccination with idiotype pulsed antigen-presenting cells post-transplant was associated with improved progression-free survival as compared to a historical control cohort.^59^ Based on these studies, we have conducted a phase II study in which patients with myeloma underwent vaccination with DC/MM fusions in conjunction with autologous transplant.^84^ Patients underwent vaccine production during the period of pre-transplant induction therapy. A majority of the patients underwent vaccination following post-transplant hematopoietic recovery, while a small cohort underwent pre-transplant vaccination with post-transplant boosting. Vaccination was well tolerated without evidence of clinically significant autoimmunity or impact on post-transplant engraftment. While general measures of cellular immunity were depressed following transplant, a paradoxical increase in myeloma-specific T cells was observed that was further boosted following vaccination. In the cohort of patients undergoing post-transplant vaccination alone, 29% of patients achieved complete remission within the first 100 days following transplant. Following vaccination, between day 100 and 1 year post-transplant, 54% achieved complete remission, suggesting that the induction of myeloma-specific immunity was associated with the targeting of post-transplant minimal residual disease.

## IMMUNOMODULATORY THERAPY AND VACCINATION

Immunomodulatory agents such as lenalidomide and pomalidomide have demonstrated significant efficacy in patients with myeloma through a variety of mechanisms, including direct cytotoxicity, inhibition of stromal myeloma cell interactions, and impact on anti-myeloma immunity.^85^,^86^ As these agents are thought to enhance the immunologic environment in patients with myeloma, there is considerable interest in combining these agents with tumor vaccines. Lenalidomide has been shown to augment NK cell function.^87^ We demonstrated that lenalidomide decreases PD-1 expression by T cells and polarizes T cell towards a Th1 as compared to a Th2 phenotype after stimulation with DC/tumor fusions.^88^ Previous studies have demonstrated that lenalidomide enhances response to the Pneumovax vaccine.^89^ Based on these findings, a multicenter phase II randomized trial is being initiated by the Clinical Trials Network Cooperative group in which patients will receive post-transplant lenalidomide maintenance alone or in conjunction with serial vaccination with DC/myeloma fusions.

The negative co-stimulatory molecules CTLA-4 and PD-L1/PD-1 are critical mediators of tumor-mediated immune suppression and tolerance, and antibodies that block their function have become a major new area of cancer therapeutics.^22^,^90^ Blockade of CTLA-4 has demonstrated activity via activation of cell-mediated immunity and has received approval by the FDA for patients with recurrent melanoma. Blockade of PD-1 has been shown to induce durable disease regression in a subset of patients with melanoma and renal and non-small lung cancer, and is currently being studied in patients with hematological malignancies. In a phase II trial, patients with recurrent lymphoma underwent serial infusions with PD-1 antibody following autologous transplantation. Investigators are now examining the potential synergy between checkpoint blockade and vaccine therapy.

We have demonstrated that PD-L1 is strongly expressed by human myeloma cell lines as well as patient-derived samples. Increased expression of PD-1 is seen on circulating T cells in patients with active disease. We have shown that PD-1 blockade augments the efficacy of the DC/MM fusion vaccine *in vitro* as manifested by increased T cell expression of IFNγ, decreased expansion of regulatory T cells, and enhanced lysis of myeloma targets.^91^ Based on these findings, a clinical trial is now underway examining the efficacy of a PD-1 antibody alone or in conjunction with the DC/MM fusion vaccine following autologous transplantation. Preliminary results have demonstrated that post-transplant treatment with PD-1 antibody resulted in the expansion of myeloma-specific T cells and antigen-specific responses against MUC1, WT1, PRAME, NYESO, and survivin in the bone marrow and peripheral blood.^92^

## DENDRITIC CELL/TUMOR FUSION VACCINATION FOR PATIENTS WITH ACUTE MYELOID LEUKEMIA

Given the findings in myeloma, we have explored the potential role of fusion cell vaccination in patients with acute myeloid leukemia. The potential role of cellular immunotherapy in targeting acute myeloid leukemia cells is highlighted by the observation that a subset of patients are rendered disease-free following allogeneic transplantation due to the graft-versus-leukemia effect mediated by alloreactive T cells. We are conducting a clinical trial in which patients with active disease undergo collection of leukemia cells. Those patients who achieve remission subsequently undergo vaccine generation and serial vaccination. In preliminary findings, vaccination was associated with the induction of leukemia-specific immunity as manifested by the expansion of tumor-reactive lymphocytes and leukemia antigen-specific T cells. In the initial cohort of treated patients with a median age of 66, 70% have remained in remission with a mean follow-up of 3 years.

In conclusion, tumors frequently interfere with the development and function of immune responses.

Cancer immunotherapy aims to employ the power and specificity of the immune system for the treatment of malignancy. Studies exploring immune combinations are ongoing, and new immunological approaches are under development.

## Abbreviations:

Ab: antibody;
APCs: antigen-presenting cells;
CTLA-4: cytotoxic T-lymphocyte associated antigen 4;
DCs: dendritic cells;
HLA: human leukocyte antigen;
HSCT: hematopoietic stem cell transplantation;
IDO: indolamine-2,3-dioxygenase;
mAb: monoclonal antibodies;
MDSC: myeloid derived suppressor cells;
MHC: major histocompatibility complex;
MM: multiple myeloma;
PD-1: programmed death-1;
PTLD: post-transplant proliferative disorders;
TAP: transporter associated with antigen processing;
TILs: tumor-infiltrating lymphocytes;
Tregs: regulatory T cells.
